# Supplementary material for: The Evolution and Characterization of the RNA Interference Pathways in Lophotrochozoa
Source: Genome Biol Evol. 2024 May 7;16(5):evae098. doi: 10.1093/gbe/evae098 (PMC11114477; doi:10.1093/gbe/evae098)
Supplement: evae098_Supplementary_Data [file evae098_supplementary_data.zip › Supplementary_materials.zip!/selected_miRNAs/NoGe_selected_miRNAs/mirdeep_pdf_files/NMRB01002613.1_Notospermus_geniculatus_strain_Ushimado_scaffold2613,_whole_genome_shotgun_sequence_39532.pdf]

| | | |
|---|---|---|
| Provisional ID | : | NMRB01002613.1_Notospermus_geniculatus_strain_Ushimado_scaffold2613,_whole_genome_shotgun_sequence_39532 |
| Score total | : | 156656.2 |
| Score for star read(s) | : | 3.9 |
| Score for read counts | : | 156647.5 |
| Score for mfe | : | 1.9 |
| Score for randfold | : | 0 |
| Score for cons. seed | : | 3 |
| Total read count | : | 307269 |
| Mature read count | : | 307235 |
| Loop read count | : | 0 |
| Star read count | : | 34 |

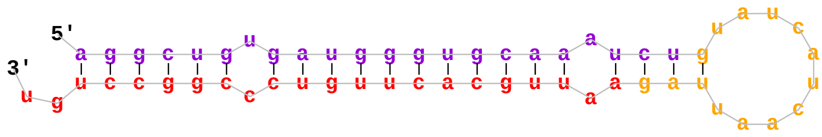


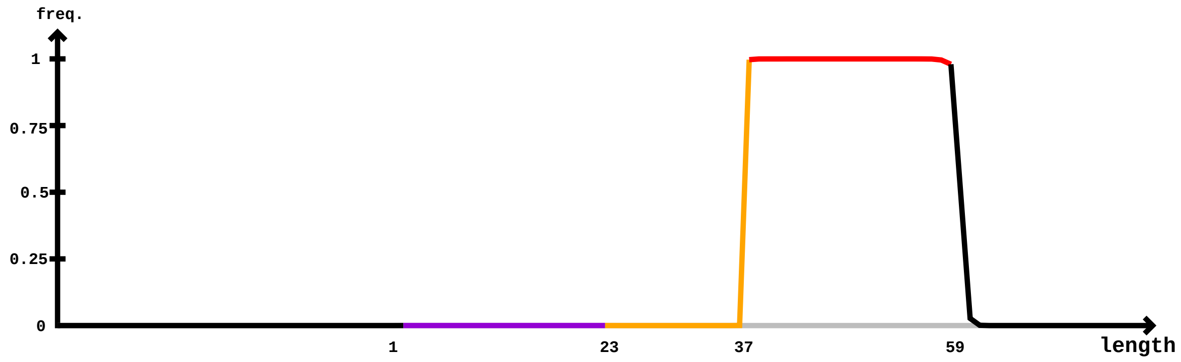


Star Mature

```
5'- uuggaguuggauuuucacuugugucauguugggcaggcugugaugggugcaaaucuguaucaucaauuagaauugcacuugucccggccuguuugauugacugcaguucauc   -3'   obs
    uuggaguuggauuuucacuugugucauguugggcaggcugugaugggugcaaaucuguaucaucaauuagaauugcacuugucccggccuguuugauugacugcaguucauc         exp
    .(((((......))))).((((((((.(((((((((((((.(((((((((((.((((..........)))).))))))))))).))))))))))))))))).)))).......   reads   mm   sample
    ..................................aggcugugauAggugcaaaucu........................................................   1       1    Fm3
    ..................................aggcugugaugggugcaaaucu........................................................   6       0    Fm3
    .....................................................................Caauugcacuugucccggccugu....................   1       1    Fm3
    ......................................................................aauugcacuugucccggc........................   3       0    Fm3
    ......................................................................aauugcacuugucccggcc.......................   12      0    Fm3
    ......................................................................aauugcacuugucccggcA.......................   1       1    Fm3
    ......................................................................aauugcacuuCucccggccu......................   1       1    Fm3
    ......................................................................aauugcacuugucccggccu......................   35      0    Fm3
    ......................................................................aauugcacuuAucccggccu......................   1       1    Fm3
    ......................................................................aauugGacuugucccggccug.....................   1       1    Fm3
    ......................................................................aauugcacuuAucccggccug.....................   3       1    Fm3
    ......................................................................aauugcacuugucccgUccug.....................   1       1    Fm3
    ......................................................................Gauugcacuugucccggccug.....................   2       1    Fm3
    ......................................................................aauugcacuugucccggcAug.....................   1       1    Fm3
    ......................................................................aauugcacuugucccggUcug.....................   4       1    Fm3
    ......................................................................aauugcacuugucccggccug.....................   882     0    Fm3
    ......................................................................aauugcacuugucccgAccug.....................   1       1    Fm3
    ......................................................................aaCugcacuugucccggccug.....................   1       1    Fm3
    ......................................................................aauugcacuGgucccggccug.....................   3       1    Fm3
    ......................................................................aauugcacuugucccggAcug.....................   8       1    Fm3
    ......................................................................aauugcacuugucccgCccug.....................   2       1    Fm3
    ......................................................................aauugcacuugucccggGcug.....................   3       1    Fm3
    ......................................................................aauugcacuuguUccggccug.....................   1       1    Fm3
    ......................................................................aauugcacuuUucccggccug.....................   1       1    Fm3
    ......................................................................Uauugcacuugucccggccug.....................   2       1    Fm3
    ......................................................................aauugcacuugucccggccuA.....................   1       1    Fm3
    ......................................................................aauugcacuugucccgCccugu....................   27      1    Fm3
    ......................................................................aauuAcacuugucccggccugu....................   2       1    Fm3
    ......................................................................aauugGacuugucccggccugu....................   4       1    Fm3
    ......................................................................aauugcacuugucUcggccugu....................   12      1    Fm3
    ......................................................................aauugcacuugucccgAccugu....................   45      1    Fm3
    ......................................................................aauugcacuugCcccggccugu....................   2       1    Fm3
    ......................................................................aauugcacuugAcccggccugu....................   2       1    Fm3
```

```
                                  Star                                Mature
uuggaguuggauuuucacuugugucauguugggcaggcugugaugggugcaaaucuguaucaucaauuagaauugcacuugucccggccuguuugauugacugcaguucauc
......................................................................aauugcacuugucccggccuAu....................   4      1   Fm3
......................................................................aauugNacuugucccggccugu....................   7      1   Fm3
......................................................................aauugcacuugucccggcAugu....................   2      1   Fm3
......................................................................aauugcacuuguGccggccugu....................   3      1   Fm3
......................................................................aauugcacCugucccggccugu....................   1      1   Fm3
......................................................................aauugcacuCgucccggccugu....................   6275   1   Fm3
......................................................................aauugUacuugucccggccugu....................   3      1   Fm3
......................................................................aauugcacuugucccggccugC....................   40     1   Fm3
......................................................................aauugcacuuguccGggccugu....................   2      1   Fm3
......................................................................aauugcacuuAucccggccugu....................   31     1   Fm3
......................................................................aauugcacuuCucccggccugu....................   15     1   Fm3
......................................................................aauugcGcuugucccggccugu....................   11     1   Fm3
......................................................................aauugcacuugucGcggccugu....................   1      1   Fm3
......................................................................aauugcacuugucccggccugA....................   58     1   Fm3
......................................................................aauugcacuuUucccggccugu....................   11     1   Fm3
......................................................................aauugcacuugucccgUccugu....................   40     1   Fm3
......................................................................aGuugcacuugucccggccugu....................   9      1   Fm3
......................................................................aauugcacuugucccggUcugu....................   71     1   Fm3
......................................................................aauCgcacuugucccggccugu....................   6      1   Fm3
......................................................................aauugcacuugucccggccugu....................   15412  0   Fm3
......................................................................aauugcacuugucccUgccugu....................   2      1   Fm3
......................................................................aauugcacuugucccggccuUu....................   1      1   Fm3
......................................................................aauugcaUuugucccggccugu....................   1      1   Fm3
......................................................................aauugcacuugucccAgccugu....................   13     1   Fm3
......................................................................aauugcacuugucAcggccugu....................   7      1   Fm3
......................................................................aauugcacuugucccggcGugu....................   1      1   Fm3
......................................................................aauugcacuugucccggccuCu....................   1      1   Fm3
......................................................................aauuUcacuugucccggccugu....................   5      1   Fm3
......................................................................aauugcacuugucccggcUugu....................   2      1   Fm3
......................................................................aauGgcacuugucccggccugu....................   1      1   Fm3
......................................................................aauugcacuugucccggccugG....................   5      1   Fm3
......................................................................Uauugcacuugucccggccugu....................   29     1   Fm3
......................................................................Cauugcacuugucccggccugu....................   3      1   Fm3
......................................................................aauugcacuugucccggAcugu....................   133    1   Fm3
......................................................................aauugcCcuugucccggccugu....................   2      1   Fm3
......................................................................aCuugcacuugucccggccugu....................   3      1   Fm3
......................................................................aUuugcacuugucccggccugu....................   4      1   Fm3
......................................................................aaGugcacuugucccggccugu....................   3      1   Fm3
......................................................................aauugcacuugucccggccCgu....................   3      1   Fm3
......................................................................Gauugcacuugucccggccugu....................   2      1   Fm3
......................................................................aauugcaAuugucccggccugu....................   1      1   Fm3
......................................................................aauugcacuuguUccggccugu....................   10     1   Fm3
......................................................................aauAgcacuugucccggccugu....................   4      1   Fm3
......................................................................aauugcUcuugucccggccugu....................   6      1   Fm3
......................................................................aauugcacuAgucccggccugu....................   6      1   Fm3
......................................................................aauugcacuugucccggGcugu....................   46     1   Fm3
......................................................................aauuCcacuugucccggccugu....................   1      1   Fm3
......................................................................aauugcacuuguccAggccugu....................   4      1   Fm3
......................................................................aauugAacuugucccggccugu....................   2      1   Fm3
......................................................................aauugcacuuguccUggccugu....................   22     1   Fm3
......................................................................aauugcacuuguAccggccugu....................   5      1   Fm3
......................................................................aaCugcacuugucccggccugu....................   5      1   Fm3
......................................................................Nauugcacuugucccggccugu....................   7      1   Fm3
......................................................................aauugcacuugucccggccuguC...................   1      1   Fm3
......................................................................aauugcacuAgucccggccuguu...................   1      1   Fm3
......................................................................aGuugcacuugucccggccuguu...................   1      1   Fm3
......................................................................aauugcacuugucccggccuguG...................   8      1   Fm3
......................................................................aauugcacuugucccgUccuguu...................   3      1   Fm3
......................................................................Uauugcacuugucccggccuguu...................   2      1   Fm3
......................................................................aauugcacuugucccggGcuguu...................   3      1   Fm3
......................................................................aauugcacuugucccggAcuguu...................   5      1   Fm3
......................................................................aauugcacuugucccggccugCu...................   3      1   Fm3
......................................................................aauugcGcuugucccggccuguu...................   1      1   Fm3
......................................................................aauugcacuugucccggUcuguu...................   9      1   Fm3
......................................................................aauugcacuuCucccggccuguu...................   1      1   Fm3
......................................................................aauugcacuugucGcggccuguu...................   1      1   Fm3
......................................................................aauugcacuuguUccggccuguu...................   1      1   Fm3
......................................................................aauugNacuugucccggccuguu...................   1      1   Fm3
......................................................................aauugcacuCgucccggccuguu...................   266    1   Fm3
......................................................................aauugcacuugucUcggccuguu...................   3      1   Fm3
```

```
                                  Star                    Mature
uuggaguuggauuuucacuugugucauguugggcaggcugugaugggugcaaaucuguaucaucaauuagaauugcacuugucccggccuguuugauugacugcaguucauc
......................................................................aauugcacuuguccAggccuguu...................   1      1    Fm3
......................................................................aauuUcacuugucccggccuguu...................   1      1    Fm3
......................................................................aauugcacuugucccgAccuguu...................   1      1    Fm3
......................................................................aauugcacuugucccggccuguu...................   1036   0    Fm3
......................................................................aauugcacuugucccggccuguA...................   912    1    Fm3
......................................................................aauugcacuugucccgCccuguu...................   1      1    Fm3
......................................................................aauugcacuuAucccggccuguu...................   3      1    Fm3
......................................................................aauugcacuuguGccggccuguu...................   1      1    Fm3
......................................................................aauugcacuuguAccggccuguu...................   1      1    Fm3
......................................................................aauugcacuugucccggccuguuA..................   4      1    Fm3
......................................................................aauugcacuCgucccggccuguuu..................   45     1    Fm3
......................................................................aauugcacuugucccggAcuguuu..................   2      1    Fm3
......................................................................aauugcacuugucccggccuguuu..................   86     0    Fm3
......................................................................aauugcacuugucccggccuguuC..................   2      1    Fm3
......................................................................aauugcacuuguccUggccuguuu..................   3      1    Fm3
......................................................................aauugcacuugucccggccuguAu..................   436    1    Fm3
......................................................................aauugcacuugucccggccuguGu..................   1      1    Fm3
......................................................................aauugcacuugucccggccuguuuU.................   3      1    Fm3
.......................................................................auugcacuugucccggccug.....................   4      0    Fm3
.......................................................................auugcacuugucccggcGugu....................   1      1    Fm3
.......................................................................auugcacuugucccggccugu....................   40     0    Fm3
.......................................................................auugcacAugucccggccugu....................   1      1    Fm3
.......................................................................auugcacuuUucccggccugu....................   4      1    Fm3
.......................................................................auugcacuugucccggccugC....................   1      1    Fm3
.......................................................................auugcacuCgucccggccugu....................   51     1    Fm3
.......................................................................auugcacuugucccggccCgu....................   1      1    Fm3
.......................................................................auugcacuugucccggccuguA...................   10     1    Fm3
.......................................................................auugcacuCgucccggccuguu...................   2      1    Fm3
.......................................................................auugcacuugucccggAcuguu...................   1      1    Fm3
.......................................................................auugcacuugucccggccuguu...................   8      0    Fm3
.......................................................................auugcacuugucccggccuguuA..................   1      1    Fm3
.......................................................................auugcacuugucccggccuguAu..................   4      1    Fm3
.......................................................................auugcacuCgucccggccuguuu..................   1      1    Fm3
........................................................................uugcacuugucccggccugC....................   3      1    Fm3
........................................................................uugcacuugucccggccugu....................   1      0    Fm3
........................................................................uugcacuugucccggccuguu...................   2      0    Fm3
.........................................................................ugcacuugucccggccugu....................   1      0    Fm3

..................................aggcugugaugggugcaaaucu........................................................   5      0    Mm3
....................................................................aAaauugcacuugucccggccuguu...................   1      1    Mm3
.....................................................................Caauugcacuugucccggccugu....................   14     1    Mm3
.....................................................................Caauugcacuugucccggccuguu...................   2      1    Mm3
......................................................................aauugcacuugucccggc........................   8      0    Mm3
......................................................................Nauugcacuugucccggc........................   1      1    Mm3
......................................................................aauugcacuugucccggcA.......................   1      1    Mm3
......................................................................aauugcacuuguccUggcc.......................   1      1    Mm3
......................................................................aauugcacuugucccggcc.......................   28     0    Mm3
......................................................................aauugcacuugucccggcU.......................   1      1    Mm3
......................................................................aauugcacuuAucccggccu......................   8      1    Mm3
......................................................................aauugcacuugucccggccA......................   2      1    Mm3
......................................................................aauugcacuugucccggcUu......................   1      1    Mm3
......................................................................aauugcacuugucccggGcu......................   1      1    Mm3
......................................................................aauugcacuugucccggccG......................   10     1    Mm3
......................................................................aauugcacuugucccgUccu......................   1      1    Mm3
......................................................................aauugcacuugucccggAcu......................   2      1    Mm3
......................................................................aauugcacuAgucccggccu......................   1      1    Mm3
......................................................................aauugcacuugucccggccu......................   379    0    Mm3
......................................................................aauugcacuugucccggccC......................   3      1    Mm3
......................................................................aauugcacuugucccggUcu......................   2      1    Mm3
......................................................................aauugcacuuCucccggccu......................   2      1    Mm3
......................................................................aauugcacuugucccgCccu......................   3      1    Mm3
......................................................................aauugcacuugucccggGcug.....................   7      1    Mm3
......................................................................aauugcacuugucccggAcug.....................   11     1    Mm3
......................................................................aGuugcacuugucccggccug.....................   1      1    Mm3
......................................................................aauugcacuugucccggccCg.....................   11     1    Mm3
......................................................................aauugAacuugucccggccug.....................   1      1    Mm3
......................................................................aauugcacuugucccggccGg.....................   13     1    Mm3
......................................................................aauugcacuuguUccggccug.....................   3      1    Mm3
......................................................................aauugcacuugucccgAccug.....................   3      1    Mm3
......................................................................aauugcacuugucccggccuA.....................   2      1    Mm3
```

| Star Mature | | | |
|---|---|---|---|
| uuggaguuggauuuucacuugugucauguugggcaggcugugaugggugcaaaucuguaucaucaauuagaauugcacuugucccggccuguuugauugacugcaguucauc | | | |
| ......................................................................aauugcacuugucccggccuC..................... | 1 | 1 | Mm3 |
| ......................................................................aauugcacuugucccgUccug..................... | 4 | 1 | Mm3 |
| ......................................................................aauugcacuugucccggccuU..................... | 5 | 1 | Mm3 |
| ......................................................................aauugcacuugucccgCccug..................... | 1 | 1 | Mm3 |
| ......................................................................aauugcacuugucccggccug..................... | 748 | 0 | Mm3 |
| ......................................................................aauugcacuuAucccggccug..................... | 1 | 1 | Mm3 |
| ......................................................................aauugcacuuUucccggccug..................... | 7 | 1 | Mm3 |
| ......................................................................aauugcacuugucccggUcug..................... | 5 | 1 | Mm3 |
| ......................................................................aauuUcacuugucccggccugu.................... | 7 | 1 | Mm3 |
| ......................................................................aauCgcacuugucccggccugu.................... | 5 | 1 | Mm3 |
| ......................................................................aauugcCcuugucccggccugu.................... | 2 | 1 | Mm3 |
| ......................................................................aauugcacuugucAcggccugu.................... | 8 | 1 | Mm3 |
| ......................................................................aauugcacuuCucccggccugu.................... | 45 | 1 | Mm3 |
| ......................................................................aauugcacuuguGccggccugu.................... | 2 | 1 | Mm3 |
| ......................................................................aUuugcacuugucccggccugu.................... | 9 | 1 | Mm3 |
| ......................................................................Nauugcacuugucccggccugu.................... | 15 | 1 | Mm3 |
| ......................................................................aauugAacuugucccggccugu.................... | 2 | 1 | Mm3 |
| ......................................................................aauugcacuuAucccggccugu.................... | 96 | 1 | Mm3 |
| ......................................................................aauugcacuugucccggAcugu.................... | 371 | 1 | Mm3 |
| ......................................................................aauugcacuugAcccggccugu.................... | 1 | 1 | Mm3 |
| ......................................................................aaGugcacuugucccggccugu.................... | 6 | 1 | Mm3 |
| ......................................................................aauugcacuugucGcggccugu.................... | 7 | 1 | Mm3 |
| ......................................................................aauugcacAugucccggccugu.................... | 5 | 1 | Mm3 |
| ......................................................................aauugcacuugucccggccugu.................... | 43909 | 0 | Mm3 |
| ......................................................................aauugNacuugucccggccugu.................... | 3 | 1 | Mm3 |
| ......................................................................aauugcacuugucccggccugG.................... | 434 | 1 | Mm3 |
| ......................................................................aauugcacuuguccUggccugu.................... | 24 | 1 | Mm3 |
| ......................................................................aauugcacuugucccggccAgu.................... | 1 | 1 | Mm3 |
| ......................................................................aauugcacGugucccggccugu.................... | 3 | 1 | Mm3 |
| ......................................................................aauugcaUuugucccggccugu.................... | 8 | 1 | Mm3 |
| ......................................................................aauuAcacuugucccggccugu.................... | 3 | 1 | Mm3 |
| ......................................................................aauugcacuuguAccggccugu.................... | 5 | 1 | Mm3 |
| ......................................................................Gauugcacuugucccggccugu.................... | 6 | 1 | Mm3 |
| ......................................................................aauugUacuugucccggccugu.................... | 10 | 1 | Mm3 |
| ......................................................................aauugcacuuUucccggccugu.................... | 115 | 1 | Mm3 |
| ......................................................................aauugcacuugucccggccugA.................... | 70 | 1 | Mm3 |
| ......................................................................aauugcacuugucccgUccugu.................... | 96 | 1 | Mm3 |
| ......................................................................aauugcacuugucccggUcugu.................... | 227 | 1 | Mm3 |
| ......................................................................aauugcacuugucccggccuCu.................... | 42 | 1 | Mm3 |
| ......................................................................aauugcacuugucccggccuUu.................... | 11 | 1 | Mm3 |
| ......................................................................aauugcaAuugucccggccugu.................... | 1 | 1 | Mm3 |
| ......................................................................aauugcacuugucccAgccugu.................... | 10 | 1 | Mm3 |
| ......................................................................aauugcUcuugucccggccugu.................... | 8 | 1 | Mm3 |
| ......................................................................aauugcacuugCcccggccugu.................... | 3 | 1 | Mm3 |
| ......................................................................aauugcacuugucccUgccugu.................... | 4 | 1 | Mm3 |
| ......................................................................aauugcacuGgucccggccugu.................... | 1 | 1 | Mm3 |
| ......................................................................aauugcacuugucccggccugC.................... | 328 | 1 | Mm3 |
| ......................................................................Uauugcacuugucccggccugu.................... | 53 | 1 | Mm3 |
| ......................................................................aCuugcacuugucccggccugu.................... | 6 | 1 | Mm3 |
| ......................................................................aauAgcacuugucccggccugu.................... | 3 | 1 | Mm3 |
| ......................................................................aauugcacuugucccggcUugu.................... | 6 | 1 | Mm3 |
| ......................................................................aauGgcacuugucccggccugu.................... | 1 | 1 | Mm3 |
| ......................................................................aauuCcacuugucccggccugu.................... | 1 | 1 | Mm3 |
| ......................................................................aauugcNcuugucccggccugu.................... | 3 | 1 | Mm3 |
| ......................................................................aauugcacuugucccggccCgu.................... | 5 | 1 | Mm3 |
| ......................................................................aauugcacuuguUccggccugu.................... | 21 | 1 | Mm3 |
| ......................................................................aauugcacuugucccCgccugu.................... | 5 | 1 | Mm3 |
| ......................................................................aNuugcacuugucccggccugu.................... | 1 | 1 | Mm3 |
| ......................................................................aaNugcacuugucccggccugu.................... | 1 | 1 | Mm3 |
| ......................................................................aauugcacuugucccggccuAu.................... | 3 | 1 | Mm3 |
| ......................................................................aauugcacuugucUcggccugu.................... | 23 | 1 | Mm3 |
| ......................................................................aauugcacuCgucccggccugu.................... | 6582 | 1 | Mm3 |
| ......................................................................aaAugcacuugucccggccugu.................... | 10 | 1 | Mm3 |
| ......................................................................aauugcaGuugucccggccugu.................... | 1 | 1 | Mm3 |
| ......................................................................aauugcacuuguccGggccugu.................... | 3 | 1 | Mm3 |
| ......................................................................aauugcacuugucccggccGgu.................... | 3 | 1 | Mm3 |
| ......................................................................aauugcacuugucccggcAugu.................... | 2 | 1 | Mm3 |
| ......................................................................aauugcacuugucccgAccugu.................... | 110 | 1 | Mm3 |
| ......................................................................aauugcacCugucccggccugu.................... | 1 | 1 | Mm3 |
| ......................................................................Cauugcacuugucccggccugu.................... | 3 | 1 | Mm3 |

Star Mature

```
uuggaguuggauuuucacuugugucauguugggcaggcugugaugggugcaaaucuguaucaucaauuagaauugcacuugucccggccuguuugauugacugcaguucauc
......................................................................aGuugcacuugucccggccugu....................  14    1  Mm3
......................................................................aauugcacuuguccAggccugu....................  3     1  Mm3
......................................................................aauugcGcuugucccggccugu....................  9     1  Mm3
......................................................................aaCugcacuugucccggccugu....................  8     1  Mm3
......................................................................aauugcacuugucccgCccugu....................  114   1  Mm3
......................................................................aauugcacuAgucccggccugu....................  5     1  Mm3
......................................................................aauugcacuugucccggGcugu....................  164   1  Mm3
......................................................................aauugcacuugucccggcAuguu...................  1     1  Mm3
......................................................................aauugcacuuAucccggccuguu...................  8     1  Mm3
......................................................................aauugcacuuUucccggccuguu...................  9     1  Mm3
......................................................................aauugUacuugucccggccuguu...................  1     1  Mm3
......................................................................aUuugcacuugucccggccuguu...................  3     1  Mm3
......................................................................aauugcacuugucccggccugAu...................  1     1  Mm3
......................................................................aauugcacuugucAcggccuguu...................  1     1  Mm3
......................................................................aauugcacuugucUcggccuguu...................  3     1  Mm3
......................................................................aGuugcacuugucccggccuguu...................  3     1  Mm3
......................................................................aauugcacuugucccggccuguC...................  2     1  Mm3
......................................................................aauugcacuugucccggAcuguu...................  41    1  Mm3
......................................................................aauugcacuugucccggccuguu...................  4307  0  Mm3
......................................................................aauugcGcuugucccggccuguu...................  5     1  Mm3
......................................................................aauugcacuuCucccggccuguu...................  4     1  Mm3
......................................................................aauugcacuugucccgAccuguu...................  17    1  Mm3
......................................................................aauugcacuCgucccggccuguu...................  861   1  Mm3
......................................................................aauugcacuugucccggccugGu...................  1     1  Mm3
......................................................................aauugcacuAgucccggccuguu...................  1     1  Mm3
......................................................................aauugcacuugucccggccuUuu...................  2     1  Mm3
......................................................................aauugcacuugucccggGcuguu...................  17    1  Mm3
......................................................................aaAugcacuugucccggccuguu...................  1     1  Mm3
......................................................................Gauugcacuugucccggccuguu...................  1     1  Mm3
......................................................................aauugcacuugucccggccuAuu...................  2     1  Mm3
......................................................................aaCugcacuugucccggccuguu...................  1     1  Mm3
......................................................................aauugcacAugucccggccuguu...................  1     1  Mm3
......................................................................aauugcacuugucccggccuguA...................  2392  1  Mm3
......................................................................aauugcacuugucccgCccuguu...................  9     1  Mm3
......................................................................aauuUcacuugucccggccuguu...................  1     1  Mm3
......................................................................aauugcacuGgucccggccuguu...................  1     1  Mm3
......................................................................Nauugcacuugucccggccuguu...................  5     1  Mm3
......................................................................aauugcacuugAcccggccuguu...................  1     1  Mm3
......................................................................aauugcacuugucccggccugCu...................  33    1  Mm3
......................................................................aauugcacuuguccUggccuguu...................  2     1  Mm3
......................................................................aauugcaGuugucccggccuguu...................  1     1  Mm3
......................................................................aauugcacuugucccggccuCuu...................  5     1  Mm3
......................................................................aauugcaUuugucccggccuguu...................  1     1  Mm3
......................................................................aauugcacuugucccggUcuguu...................  22    1  Mm3
......................................................................aauugcaAuugucccggccuguu...................  1     1  Mm3
......................................................................aauCgcacuugucccggccuguu...................  1     1  Mm3
......................................................................Uauugcacuugucccggccuguu...................  8     1  Mm3
......................................................................aauugcacuugucccggccuguG...................  20    1  Mm3
......................................................................aauugAacuugucccggccuguu...................  1     1  Mm3
......................................................................aauugcacuugucccAgccuguu...................  2     1  Mm3
......................................................................aauugcacuuguccAggccuguu...................  1     1  Mm3
......................................................................aauugcacuugucccgUccuguu...................  9     1  Mm3
......................................................................aauugcacuugucccggccuguuG..................  5     1  Mm3
......................................................................aauugcacuuguGccggccuguuu..................  1     1  Mm3
......................................................................aauugcacuugucAcggccuguuu..................  1     1  Mm3
......................................................................aUuugcacuugucccggccuguuu..................  1     1  Mm3
......................................................................aauugcacuuAucccggccuguuu..................  1     1  Mm3
......................................................................aauugcacuugucccgAccuguuu..................  4     1  Mm3
......................................................................aauugcacuCgucccggccuguuu..................  220   1  Mm3
......................................................................aauugcacuugucccggccugAuu..................  1     1  Mm3
......................................................................aaGugcacuugucccggccuguuu..................  1     1  Mm3
......................................................................aauugcacuugucccggccugCuu..................  1     1  Mm3
......................................................................aauugcacuugucccggUcuguuu..................  3     1  Mm3
......................................................................aauugcacuugucccggccuguuA..................  36    1  Mm3
......................................................................aauugcacuugucccggccuguuC..................  1     1  Mm3
......................................................................aauugcacuugucccggccuguuu..................  846   0  Mm3
......................................................................aauugcacuugucccggccuguAu..................  931   1  Mm3
......................................................................aauugcacuugucccgCccuguuu..................  1     1  Mm3
......................................................................aauugcacuugucccggGcuguuu..................  2     1  Mm3
......................................................................aaCugcacuugucccggccuguuu..................  1     1  Mm3
```

```
                                 Star                                 Mature
uuggaguuggauuuucacuugugucauguuggcaggcugugaugggugcaaaucuguaucaucaauuagaauugcacuugucccggccuguuugauugacugcaguucauc
.....................................................................aauugcacuugucccggccuguGu..................  2      1    Mm3
.....................................................................aauugcacuugucccggAcuguu...................  7      1    Mm3
.....................................................................aauugcacuuUucccggccuguu...................  5      1    Mm3
.....................................................................aauugcacuugucccggccuguuuU.................  55     1    Mm3
......................................................................auuAcacuugucccggccu......................  1      1    Mm3
......................................................................auugcacuugucccggccCg.....................  1      1    Mm3
......................................................................auugcacuugucccggccug.....................  1      0    Mm3
......................................................................auugcacuugCcccggccugu....................  1      1    Mm3
......................................................................auugcacuugucccggccugu....................  102    0    Mm3
......................................................................auugcacuCgucccggccugu....................  19     1    Mm3
......................................................................auugcacuugucccggccuUu....................  2      1    Mm3
......................................................................auugcacuuUucccggccugu....................  3      1    Mm3
......................................................................auugcacuugucccggccugC....................  1      1    Mm3
......................................................................auugcaGuugucccggccugu....................  1      1    Mm3
......................................................................auugcacCugucccggccugu....................  1      1    Mm3
......................................................................auugcacuCgucccggccuguu...................  2      1    Mm3
......................................................................auugcacuugucccggccuguu...................  14     0    Mm3
......................................................................auugcacuuUucccggccuguu...................  1      1    Mm3
......................................................................auugcacuugucccggccuguA...................  17     1    Mm3
......................................................................auugcacuugucccggccuguAu..................  15     1    Mm3
......................................................................auugcacuugucccggccuguuu..................  1      0    Mm3
.......................................................................uugcacuugucccggccug.....................  2      0    Mm3
.......................................................................uugcacuugucccggccugu....................  2      0    Mm3
.......................................................................uugcacuugucccggccuguu...................  1      0    Mm3
........................................................................ugcacuugucccggccug.....................  1      0    Mm3
........................................................................ugcacuugucccggccuguu...................  1      0    Mm3
.........................................................................gcacuugucccggccugu....................  8      0    Mm3
.........................................................................gcacuugucccggccuguu...................  1      0    Mm3
.........................................................................gcacuugucccggccuguA...................  1      1    Mm3
...........................................................................acuugucccggccuguuu..................  1      0    Mm3
...........................................................................acuugucccggccuguAu..................  1      1    Mm3

.................................aggcugugaugggugcaaaucu........................................................  2      0    Fm1
.................................aggcugugaugggugcaCaucu........................................................  1      1    Fm1
....................................................ucuguaucaucaauuagaauugcacuugucccggccugu....................  1      0    Fm1
....................................................................Caauugcacuugucccggccug.....................  3      1    Fm1
....................................................................Caauugcacuugucccggccugu....................  15     1    Fm1
....................................................................Caauugcacuugucccggccuguu...................  2      1    Fm1
.....................................................................aauugcacuugucccggcU.......................  5      1    Fm1
.....................................................................aauugcacuuAucccggccu......................  6      1    Fm1
.....................................................................aauugcacuugucccggccu......................  107    0    Fm1
.....................................................................aauugcacuugucccggccG......................  8      1    Fm1
.....................................................................aauugcacuugucccggAcu......................  1      1    Fm1
.....................................................................aauugcGcuugucccggccug.....................  1      1    Fm1
.....................................................................aauugcacuAgucccggccug.....................  1      1    Fm1
.....................................................................aauugcacuugucccggccGg.....................  13     1    Fm1
.....................................................................aauugcacuugucccggccuU.....................  5      1    Fm1
.....................................................................aauugcacuugucccggccug.....................  389    0    Fm1
.....................................................................aaGugcacuugucccggccug.....................  1      1    Fm1
.....................................................................aauugcacuugucccggUcug.....................  2      1    Fm1
.....................................................................aauugcacuugucccggGcug.....................  1      1    Fm1
.....................................................................Uauugcacuugucccggccug.....................  1      1    Fm1
.....................................................................aauugcacuugucccgUccug.....................  2      1    Fm1
.....................................................................aauugcacuugucccggAcug.....................  1      1    Fm1
.....................................................................aauugcacuugucccggccuA.....................  3      1    Fm1
.....................................................................aauugcacuuguAccggccug.....................  1      1    Fm1
.....................................................................aauugcacuugucccgAccug.....................  1      1    Fm1
.....................................................................aauugcacuuguGccggccugu....................  1      1    Fm1
.....................................................................aauuUcacuugucccggccugu....................  1      1    Fm1
.....................................................................aauugcacAugucccggccugu....................  3      1    Fm1
.....................................................................aauugcacuugucccAgccugu....................  4      1    Fm1
.....................................................................aauugNacuugucccggccugu....................  2      1    Fm1
.....................................................................aauugcacuugucccgAccugu....................  30     1    Fm1
.....................................................................aauugcacuuguccUggccugu....................  6      1    Fm1
.....................................................................aauugcacuugucccggccGgu....................  1      1    Fm1
.....................................................................aaGugcacuugucccggccugu....................  1      1    Fm1
.....................................................................aauugcacuugucccggccugA....................  18     1    Fm1
.....................................................................Cauugcacuugucccggccugu....................  2      1    Fm1
.....................................................................aauugcacuugucccggccugu....................  12699  0    Fm1
.....................................................................aaCugcacuugucccggccugu....................  1      1    Fm1
```

Star Mature

| | | | |
|---|---|---|---|
| uuggaguuggauuuucacuugugucauguugggcaggcugugaugggugcaaaucuguaucaucaauuagaauugcacuugucccggccuguuugauugacugcaguucauc | | | |
| ......................................................................aauugcacuugucGcggccugu.................... | 4 | 1 | Fm1 |
| ......................................................................aauAgcacuugucccggccugu.................... | 5 | 1 | Fm1 |
| ......................................................................aauugcacuugucccggUcugu.................... | 76 | 1 | Fm1 |
| ......................................................................aauugUacuugucccggccugu.................... | 4 | 1 | Fm1 |
| ......................................................................aauugcacuugucccggccugG.................... | 256 | 1 | Fm1 |
| ......................................................................aGuugcacuugucccggccugu.................... | 3 | 1 | Fm1 |
| ......................................................................aauugcacuuguUccggccugu.................... | 9 | 1 | Fm1 |
| ......................................................................aauugcacuuguAccggccugu.................... | 2 | 1 | Fm1 |
| ......................................................................aCuugcacuugucccggccugu.................... | 1 | 1 | Fm1 |
| ......................................................................aauugcacuugucAcggccugu.................... | 4 | 1 | Fm1 |
| ......................................................................aauugcacuugucccggccuUu.................... | 4 | 1 | Fm1 |
| ......................................................................aauugcacuugucccggccCgu.................... | 3 | 1 | Fm1 |
| ......................................................................Uauugcacuugucccggccugu.................... | 13 | 1 | Fm1 |
| ......................................................................Nauugcacuugucccggccugu.................... | 8 | 1 | Fm1 |
| ......................................................................aauugcacuugucccggcGugu.................... | 2 | 1 | Fm1 |
| ......................................................................aauugcacuugucccggccugC.................... | 38 | 1 | Fm1 |
| ......................................................................aauugcGcuugucccggccugu.................... | 6 | 1 | Fm1 |
| ......................................................................aauCgcacuugucccggccugu.................... | 6 | 1 | Fm1 |
| ......................................................................aauugAacuugucccggccugu.................... | 3 | 1 | Fm1 |
| ......................................................................aauugcacuugucccggcUugu.................... | 2 | 1 | Fm1 |
| ......................................................................aauugcacuugucccggAcugu.................... | 112 | 1 | Fm1 |
| ......................................................................aauugcacuCgucccggccugu.................... | 4202 | 1 | Fm1 |
| ......................................................................aauugcacuugCcccggccugu.................... | 1 | 1 | Fm1 |
| ......................................................................Gauugcacuugucccggccugu.................... | 2 | 1 | Fm1 |
| ......................................................................aauuAcacuugucccggccugu.................... | 2 | 1 | Fm1 |
| ......................................................................aaNugcacuugucccggccugu.................... | 1 | 1 | Fm1 |
| ......................................................................aauugcacuugucccggccuAu.................... | 1 | 1 | Fm1 |
| ......................................................................aauugcaUuugucccggccugu.................... | 2 | 1 | Fm1 |
| ......................................................................aauuCcacuugucccggccugu.................... | 2 | 1 | Fm1 |
| ......................................................................aauugcacuuguccAggccugu.................... | 1 | 1 | Fm1 |
| ......................................................................aauugcacuugucccggccuCu.................... | 12 | 1 | Fm1 |
| ......................................................................aauugcacuugucccggccAgu.................... | 1 | 1 | Fm1 |
| ......................................................................aauugcacuugucccggcAugu.................... | 3 | 1 | Fm1 |
| ......................................................................aauugcacuugucccgCccugu.................... | 30 | 1 | Fm1 |
| ......................................................................aauugcacuugucUcggccugu.................... | 9 | 1 | Fm1 |
| ......................................................................aaAugcacuugucccggccugu.................... | 5 | 1 | Fm1 |
| ......................................................................aauugcacuugucccgUccugu.................... | 25 | 1 | Fm1 |
| ......................................................................aauugcacuuAucccggccugu.................... | 20 | 1 | Fm1 |
| ......................................................................aauugcacCugucccggccugu.................... | 2 | 1 | Fm1 |
| ......................................................................aauugcacuuUucccggccugu.................... | 7 | 1 | Fm1 |
| ......................................................................aauugcacuugucccggGcugu.................... | 47 | 1 | Fm1 |
| ......................................................................aauugcUcuugucccggccugu.................... | 3 | 1 | Fm1 |
| ......................................................................aauugcacuuCucccggccugu.................... | 10 | 1 | Fm1 |
| ......................................................................aauugNacuugucccggccuguu................... | 1 | 1 | Fm1 |
| ......................................................................aauugcacuugucccggccuguC................... | 1 | 1 | Fm1 |
| ......................................................................aauugcacuugucccggccuguG................... | 31 | 1 | Fm1 |
| ......................................................................aauugcacuuAucccggccuguu................... | 9 | 1 | Fm1 |
| ......................................................................aauugcacuugucccgCccuguu................... | 2 | 1 | Fm1 |
| ......................................................................aUuugcacuugucccggccuguu................... | 1 | 1 | Fm1 |
| ......................................................................aauugcacuugucccUgccuguu................... | 1 | 1 | Fm1 |
| ......................................................................aauugcacuugucccggccugCu................... | 2 | 1 | Fm1 |
| ......................................................................aauugcacuCgucccggccuguu................... | 1117 | 1 | Fm1 |
| ......................................................................aauugcacuugucUcggccuguu................... | 1 | 1 | Fm1 |
| ......................................................................aauugcacuugucccggGcuguu................... | 5 | 1 | Fm1 |
| ......................................................................aauugcacuugucccggUcuguu................... | 12 | 1 | Fm1 |
| ......................................................................aauugcacuuUucccggccuguu................... | 3 | 1 | Fm1 |
| ......................................................................aauugcacuugucccgAccuguu................... | 4 | 1 | Fm1 |
| ......................................................................aauCgcacuugucccggccuguu................... | 1 | 1 | Fm1 |
| ......................................................................aauugcacuugucccgUccuguu................... | 6 | 1 | Fm1 |
| ......................................................................aauugcacuugucccggAcuguu................... | 26 | 1 | Fm1 |
| ......................................................................aauugcGcuugucccggccuguu................... | 1 | 1 | Fm1 |
| ......................................................................Nauugcacuugucccggccuguu................... | 1 | 1 | Fm1 |
| ......................................................................aauuAcacuugucccggccuguu................... | 1 | 1 | Fm1 |
| ......................................................................aauugUacuugucccggccuguu................... | 2 | 1 | Fm1 |
| ......................................................................aauugcacuugucccggccuAuu................... | 1 | 1 | Fm1 |
| ......................................................................aauugcacuuCucccggccuguu................... | 2 | 1 | Fm1 |
| ......................................................................aauugcacuugucccggccuguA................... | 1163 | 1 | Fm1 |
| ......................................................................aauugcacuugucccggccuUuu................... | 1 | 1 | Fm1 |
| ......................................................................aauugcacuuguUccggccuguu................... | 1 | 1 | Fm1 |
| ......................................................................aauugcacuugucccggccuCuu................... | 1 | 1 | Fm1 |

Star Mature

| | | | |
|---|---|---|---|
| uuggaguuggauuuucacuugugucauguugggcaggcugugaugggugcaaaucuguaucaucaauuagaauugcacuugucccggccuguuugauugacugcaguucauc | | | |
| ......................................................................aauugcacuugucccggccuguu................... | 1931 | 0 | Fm1 |
| ......................................................................aauugcacuugucccggccuguuA.................. | 23 | 1 | Fm1 |
| ......................................................................aauugcacuugucccgCccuguuu.................. | 2 | 1 | Fm1 |
| ......................................................................aauugcacuuAucccggccuguuu.................. | 1 | 1 | Fm1 |
| ......................................................................Nauugcacuugucccggccuguuu.................. | 1 | 1 | Fm1 |
| ......................................................................aauugcacuugucccggAcuguuu.................. | 6 | 1 | Fm1 |
| ......................................................................aauugcacuCgucccggccuguuu.................. | 357 | 1 | Fm1 |
| ......................................................................aauugcacuugucccggccuguAu.................. | 283 | 1 | Fm1 |
| ......................................................................aauugcacuugucccgUccuguuu.................. | 1 | 1 | Fm1 |
| ......................................................................aauugcacuugucccggccugCuu.................. | 1 | 1 | Fm1 |
| ......................................................................aauugcacuugucccggUcuguuu.................. | 3 | 1 | Fm1 |
| ......................................................................aauugcacuuUucccggccuguuu.................. | 1 | 1 | Fm1 |
| ......................................................................aauugcacuugucccgAccuguuu.................. | 1 | 1 | Fm1 |
| ......................................................................aauugcacuugucAcggccuguuu.................. | 1 | 1 | Fm1 |
| ......................................................................aauugcacuugucccggGcuguuu.................. | 2 | 1 | Fm1 |
| ......................................................................aaCugcacuugucccggccuguuu.................. | 1 | 1 | Fm1 |
| ......................................................................aauugcacuugucccggccuguuu.................. | 434 | 0 | Fm1 |
| ......................................................................aauugcacuugucccggccuguuG.................. | 2 | 1 | Fm1 |
| ......................................................................aauugcacuugucccggccuguuuU................. | 29 | 1 | Fm1 |
| ......................................................................aauugcacuugucccggccuguuuA................. | 1 | 1 | Fm1 |
| .......................................................................auugcacuugucccggGcugu.................... | 1 | 1 | Fm1 |
| .......................................................................auugcacuugucccggccugu.................... | 28 | 0 | Fm1 |
| .......................................................................auugcacuuUucccggccugu.................... | 1 | 1 | Fm1 |
| .......................................................................auugcacuCgucccggccugu.................... | 8 | 1 | Fm1 |
| .......................................................................auugcacuugucccggccugG.................... | 1 | 1 | Fm1 |
| .......................................................................auugcacuCgucccggccuguu................... | 3 | 1 | Fm1 |
| .......................................................................auugcacuuguccUggccuguu................... | 1 | 1 | Fm1 |
| .......................................................................auugcacuugucccggccuguu................... | 4 | 0 | Fm1 |
| .......................................................................auugcacuugucccggccuguA................... | 11 | 1 | Fm1 |
| .......................................................................auugcacuugucccggccuguAu.................. | 5 | 1 | Fm1 |
| .......................................................................auugcacuCgucccggccuguuu.................. | 2 | 1 | Fm1 |
| ........................................................................uugcacuCgucccggccugu.................... | 2 | 1 | Fm1 |
| ..........................................................................gcacuugucccggccugu.................... | 4 | 0 | Fm1 |
| ......................................................................aauugcacuugucccggccG...................... | 4 | 1 | Fm2 |
| ......................................................................aauugcacuugucccggAcu...................... | 1 | 1 | Fm2 |
| ......................................................................aauugcacuugucccggccA...................... | 1 | 1 | Fm2 |
| ......................................................................aauugcacuuAucccggccu...................... | 1 | 1 | Fm2 |
| ......................................................................aauugcacuuCucccggccu...................... | 1 | 1 | Fm2 |
| ......................................................................aauugcacuugucccggccu...................... | 56 | 0 | Fm2 |
| ......................................................................aauugcacuugucccggccGg..................... | 8 | 1 | Fm2 |
| ......................................................................aauugcacuugucccggAcug..................... | 1 | 1 | Fm2 |
| ......................................................................aauugcacuugucccggUcug..................... | 1 | 1 | Fm2 |
| ......................................................................aauugcacuugucccggccuA..................... | 1 | 1 | Fm2 |
| ......................................................................aauugcacuugucccggccug..................... | 123 | 0 | Fm2 |
| ......................................................................aauugcacuugucccgUccug..................... | 1 | 1 | Fm2 |
| ......................................................................aauugcacuuCucccggccug..................... | 1 | 1 | Fm2 |
| ......................................................................aauugcacuCgucccggccugu.................... | 367 | 1 | Fm2 |
| ......................................................................aauugcacuugucccggUcugu.................... | 7 | 1 | Fm2 |
| ......................................................................aauugcacuugucccgUccugu.................... | 1 | 1 | Fm2 |
| ......................................................................aGuugcacuugucccggccugu.................... | 1 | 1 | Fm2 |
| ......................................................................aauugcacuugucccggccugu.................... | 1878 | 0 | Fm2 |
| ......................................................................aauugcacuugucccggccuCu.................... | 3 | 1 | Fm2 |
| ......................................................................aauugcacuuAucccggccugu.................... | 5 | 1 | Fm2 |
| ......................................................................aauugcacuugucccggccugC.................... | 6 | 1 | Fm2 |
| ......................................................................aauugcacuuCucccggccugu.................... | 2 | 1 | Fm2 |
| ......................................................................Cauugcacuugucccggccugu.................... | 1 | 1 | Fm2 |
| ......................................................................aauugcacuugucccggccugA.................... | 5 | 1 | Fm2 |
| ......................................................................aauugcacuugucccggAcugu.................... | 18 | 1 | Fm2 |
| ......................................................................aauugGacuugucccggccugu.................... | 1 | 1 | Fm2 |
| ......................................................................aauugcGcuugucccggccugu.................... | 5 | 1 | Fm2 |
| ......................................................................aauugcacuugAcccggccugu.................... | 1 | 1 | Fm2 |
| ......................................................................Nauugcacuugucccggccugu.................... | 1 | 1 | Fm2 |
| ......................................................................aauugcacuugucccggccugG.................... | 34 | 1 | Fm2 |
| ......................................................................Uauugcacuugucccggccugu.................... | 11 | 1 | Fm2 |
| ......................................................................aauugcacuugucccggccuUu.................... | 1 | 1 | Fm2 |
| ......................................................................aauugcacuugucccgAccugu.................... | 3 | 1 | Fm2 |
| ......................................................................aUuugcacuugucccggccugu.................... | 1 | 1 | Fm2 |
| ......................................................................aauugcacuugucccggGcugu.................... | 3 | 1 | Fm2 |
| ......................................................................aauugcacuugucccgCccugu.................... | 1 | 1 | Fm2 |

**Star** **Mature**

| | | | |
|---|---|---|---|
| uuggaguuggauuuucacuugugucauguugggcaggcugugaugggugcaaaucuguaucaucaauuagaauugcacuugucccggccuguuugauugacugcaguucauc | | | |
| ......................................................................aauugcacuugucccggccuguG................... | 1 | 1 | Fm2 |
| ......................................................................aauugcacuugucccgCccuguu................... | 1 | 1 | Fm2 |
| ......................................................................aauugcacuugucccUgccuguu................... | 1 | 1 | Fm2 |
| ......................................................................aauugcacuugucccggccuUuu................... | 1 | 1 | Fm2 |
| ......................................................................aauugcacuugucccggGcuguu................... | 2 | 1 | Fm2 |
| ......................................................................aauugcaUuugucccggccuguu................... | 1 | 1 | Fm2 |
| ......................................................................aauugcacuuUucccggccuguu................... | 1 | 1 | Fm2 |
| ......................................................................aauugcacuugucccggccugCu................... | 3 | 1 | Fm2 |
| ......................................................................aauugcacuugucccggccuguA................... | 362 | 1 | Fm2 |
| ......................................................................aauugcacuugucccgUccuguu................... | 2 | 1 | Fm2 |
| ......................................................................aauugcacuugucccggUcuguu................... | 1 | 1 | Fm2 |
| ......................................................................aauugcacuugucccggAcuguu................... | 3 | 1 | Fm2 |
| ......................................................................Cauugcacuugucccggccuguu................... | 2 | 1 | Fm2 |
| ......................................................................aauugcacuCgucccggccuguu................... | 209 | 1 | Fm2 |
| ......................................................................aauugcacuugucccggccuguu................... | 369 | 0 | Fm2 |
| ......................................................................aauugcacuugucccggccuguAu.................. | 47 | 1 | Fm2 |
| ......................................................................Nauugcacuugucccggccuguuu.................. | 1 | 1 | Fm2 |
| ......................................................................aauugcacuugucccggccuguuA.................. | 46 | 1 | Fm2 |
| ......................................................................aauugcacuugucccggccuguuG.................. | 13 | 1 | Fm2 |
| ......................................................................aUuugcacuugucccggccuguuu.................. | 1 | 1 | Fm2 |
| ......................................................................aauugcacuugucGcggccuguuu.................. | 1 | 1 | Fm2 |
| ......................................................................aauugcacuugucccggAcuguuu.................. | 2 | 1 | Fm2 |
| ......................................................................aauugcacuCgucccggccuguuu.................. | 105 | 1 | Fm2 |
| ......................................................................aauugcacuugucccggccuguuu.................. | 247 | 0 | Fm2 |
| ......................................................................aauugcacuugucccggccuguuuU................. | 63 | 1 | Fm2 |
| ......................................................................aauugcacuugucccggccuguuug................. | 1 | 0 | Fm2 |
| .......................................................................auugcacuugucccggccugu.................... | 4 | 0 | Fm2 |
| .......................................................................auugcacuugucccggccuguu................... | 1 | 0 | Fm2 |
| .......................................................................auugcacuugucccggccuguuu.................. | 2 | 0 | Fm2 |
| .......................................................................auugcacuugucccggccuUuuu.................. | 1 | 1 | Fm2 |
| ..................................aggcugugaugggugcaaaucu........................................................ | 12 | 0 | Mm1 |
| .....................................................................Caauugcacuugucccggccugu.................... | 4 | 1 | Mm1 |
| ......................................................................aauugcacuugucccggcc....................... | 25 | 0 | Mm1 |
| ......................................................................aauugcacuugucccggGc....................... | 1 | 1 | Mm1 |
| ......................................................................aauugcacuugucAcggcc....................... | 1 | 1 | Mm1 |
| ......................................................................aauugcacuugucccggUcu...................... | 1 | 1 | Mm1 |
| ......................................................................aauugcacuuAucccggccu...................... | 2 | 1 | Mm1 |
| ......................................................................aauugcacuAgucccggccu...................... | 1 | 1 | Mm1 |
| ......................................................................aauugcacuugucccggAcu...................... | 1 | 1 | Mm1 |
| ......................................................................aauugcacuugucccgAccu...................... | 2 | 1 | Mm1 |
| ......................................................................aauugcacuugucccggccu...................... | 140 | 0 | Mm1 |
| ......................................................................aauugcacuugucccggcUu...................... | 1 | 1 | Mm1 |
| ......................................................................aauugcacuugucccggccuA..................... | 6 | 1 | Mm1 |
| ......................................................................aauugcacuugucccggccuU..................... | 2 | 1 | Mm1 |
| ......................................................................aauugcacuugucccggccug..................... | 527 | 0 | Mm1 |
| ......................................................................aauugcacuuAucccggccug..................... | 1 | 1 | Mm1 |
| ......................................................................Uauugcacuugucccggccug..................... | 2 | 1 | Mm1 |
| ......................................................................aauugcacuugucccgCccug..................... | 1 | 1 | Mm1 |
| ......................................................................aauugcacuugucccgAccug..................... | 2 | 1 | Mm1 |
| ......................................................................aauugcacuugucccgUccug..................... | 1 | 1 | Mm1 |
| ......................................................................aauugcacuugucAcggccug..................... | 1 | 1 | Mm1 |
| ......................................................................aauugcacuAgucccggccug..................... | 1 | 1 | Mm1 |
| ......................................................................aauugcacuugucccggGcug..................... | 5 | 1 | Mm1 |
| ......................................................................aauugcacuugucccggUcug..................... | 2 | 1 | Mm1 |
| ......................................................................aauugcacuugucccggAcug..................... | 2 | 1 | Mm1 |
| ......................................................................aauugcacuuguccUggccugu.................... | 7 | 1 | Mm1 |
| ......................................................................aauugcacuugucccggccugC.................... | 36 | 1 | Mm1 |
| ......................................................................aauugcacuugucAcggccugu.................... | 5 | 1 | Mm1 |
| ......................................................................aauugcacuugucccggAcugu.................... | 243 | 1 | Mm1 |
| ......................................................................aauCgcacuugucccggccugu.................... | 5 | 1 | Mm1 |
| ......................................................................aauugcacuugucccggccugG.................... | 77 | 1 | Mm1 |
| ......................................................................aUuugcacuugucccggccugu.................... | 12 | 1 | Mm1 |
| ......................................................................aauugcCcuugucccggccugu.................... | 1 | 1 | Mm1 |
| ......................................................................aauugcacuugucUcggccugu.................... | 7 | 1 | Mm1 |
| ......................................................................aauugcacuugGcccggccugu.................... | 1 | 1 | Mm1 |
| ......................................................................aauugcacuugucccgAccugu.................... | 96 | 1 | Mm1 |
| ......................................................................Nauugcacuugucccggccugu.................... | 16 | 1 | Mm1 |
| ......................................................................aauugcacuugucGcggccugu.................... | 1 | 1 | Mm1 |
| ......................................................................aauugcacCugucccggccugu.................... | 3 | 1 | Mm1 |

```
                                  Star                                 Mature
uuggaguuggauuuucacuugugucauguugggcaggcugugaugggugcaaaucuguaucaucaauuagaauugcacuugucccggccuguuugauugacugcaguucauc
......................................................................aauugcacuuAucccggccugu....................   35     1   Mm1
......................................................................aauugcacuugucccggccuCu....................   1      1   Mm1
......................................................................aauugcacuugucccUgccugu....................   4      1   Mm1
......................................................................aauugAacuugucccggccugu....................   2      1   Mm1
......................................................................aauuUcacuugucccggccugu....................   2      1   Mm1
......................................................................aauugUacuugucccggccugu....................   4      1   Mm1
......................................................................aauugcUcuugucccggccugu....................   9      1   Mm1
......................................................................aauugcacuugucccggccuUu....................   6      1   Mm1
......................................................................aauugcacuuguGccggccugu....................   1      1   Mm1
......................................................................Gauugcacuugucccggccugu....................   7      1   Mm1
......................................................................aauugcGcuugucccggccugu....................   35     1   Mm1
......................................................................aauugcaAuugucccggccugu....................   2      1   Mm1
......................................................................aauugcacuuguccGggccugu....................   3      1   Mm1
......................................................................aauugGacuugucccggccugu....................   1      1   Mm1
......................................................................aaGugcacuugucccggccugu....................   5      1   Mm1
......................................................................aauugcacuugAcccggccugu....................   3      1   Mm1
......................................................................aauugcacuugucccggcGugu....................   1      1   Mm1
......................................................................aauAgcacuugucccggccugu....................   2      1   Mm1
......................................................................aaAugcacuugucccggccugu....................   9      1   Mm1
......................................................................aauugcacuugucccggccGgu....................   1      1   Mm1
......................................................................aauugcNcuugucccggccugu....................   2      1   Mm1
......................................................................aauugcacGugucccggccugu....................   1      1   Mm1
......................................................................aauugcacuGgucccggccugu....................   2      1   Mm1
......................................................................aauugcacuugucccAgccugu....................   1      1   Mm1
......................................................................aauugcacuuUucccggccugu....................   24     1   Mm1
......................................................................Cauugcacuugucccggccugu....................   5      1   Mm1
......................................................................aauugcacuugucccggccuAu....................   6      1   Mm1
......................................................................aauugcacuugucccggccCgu....................   2      1   Mm1
......................................................................aauuCcacuugucccggccugu....................   4      1   Mm1
......................................................................aauugcacuugucccggcAugu....................   3      1   Mm1
......................................................................aauugcacuugucccgUccugu....................   49     1   Mm1
......................................................................aauugcacuugucccggccAgu....................   1      1   Mm1
......................................................................aauGgcacuugucccggccugu....................   1      1   Mm1
......................................................................Uauugcacuugucccggccugu....................   54     1   Mm1
......................................................................aGuugcacuugucccggccugu....................   16     1   Mm1
......................................................................aauugcacuugucccggUcugu....................   181    1   Mm1
......................................................................aauugcacuugucccggccugA....................   25     1   Mm1
......................................................................aauuAcacuugucccggccugu....................   1      1   Mm1
......................................................................aauugcacuCgucccggccugu....................   5675   1   Mm1
......................................................................aauugcacuugucccggGcugu....................   106    1   Mm1
......................................................................aauugcacAugucccggccugu....................   2      1   Mm1
......................................................................aauugcaUuugucccggccugu....................   7      1   Mm1
......................................................................aauugcacuugucccgCccugu....................   70     1   Mm1
......................................................................aauugcaGuugucccggccugu....................   1      1   Mm1
......................................................................aauugcacuugCcccggccugu....................   8      1   Mm1
......................................................................aCuugcacuugucccggccugu....................   1      1   Mm1
......................................................................aauugcacuuCucccggccugu....................   27     1   Mm1
......................................................................aauugcacuuguccAggccugu....................   2      1   Mm1
......................................................................aauugcacuuguUccggccugu....................   4      1   Mm1
......................................................................aauugcacuugucccggcUugu....................   4      1   Mm1
......................................................................aauugNacuugucccggccugu....................   3      1   Mm1
......................................................................aauugcacuugucccggccugu....................   29917  0   Mm1
......................................................................aauugcacuugucccggccCguu...................   1      1   Mm1
......................................................................aauugcacuugucAcggccuguu...................   1      1   Mm1
......................................................................aauugcacuugucccggccuguG...................   6      1   Mm1
......................................................................aauugcacuCgucccggccuguu...................   400    1   Mm1
......................................................................aCuugcacuugucccggccuguu...................   1      1   Mm1
......................................................................aauugcacuuguUccggccuguu...................   1      1   Mm1
......................................................................aauugcacuugucccgUccuguu...................   4      1   Mm1
......................................................................Cauugcacuugucccggccuguu...................   1      1   Mm1
......................................................................aauugcGcuugucccggccuguu...................   1      1   Mm1
......................................................................aauugcacuugucccggccuguu...................   1742   0   Mm1
......................................................................aauugcacuugucccggccuguA...................   1363   1   Mm1
......................................................................aGuugcacuugucccggccuguu...................   3      1   Mm1
......................................................................aauugcaUuugucccggccuguu...................   1      1   Mm1
......................................................................aauugcacuuAucccggccuguu...................   2      1   Mm1
......................................................................aauAgcacuugucccggccuguu...................   2      1   Mm1
......................................................................aauugcacuugucccggccugAu...................   1      1   Mm1
......................................................................Uauugcacuugucccggccuguu...................   3      1   Mm1
......................................................................aaAugcacuugucccggccuguu...................   1      1   Mm1
```

```
                                  Star                                Mature
uuggaguuggauuuucacuugugucauguugggcaggcugugaugggugcaaaucuguaucaucaauuagaauugcacuugucccggccuguuugauugacugcaguucauc
......................................................................aauugcacuugucccgCccuguu...................   7     1   Mm1
......................................................................aauugcacuuCucccggccuguu...................   4     1   Mm1
......................................................................aauugNacuugucccggccuguu...................   1     1   Mm1
......................................................................aauugcacuugucccggUcuguu...................   5     1   Mm1
......................................................................aauugcacuuguccAggccuguu...................   1     1   Mm1
......................................................................aauugcacuugucUcggccuguu...................   1     1   Mm1
......................................................................aauugcacuugucccgAccuguu...................   3     1   Mm1
......................................................................aauugcacuugucccggGcuguu...................   4     1   Mm1
......................................................................aauugcacuugucccggAcuguu...................   19    1   Mm1
......................................................................aauugcacuCgucccggccuguuu..................   75    1   Mm1
......................................................................aauugcacuuguccUggccuguuu..................   7     1   Mm1
......................................................................aauugcacuugucccggUcuguuu..................   1     1   Mm1
......................................................................aauugcacuugucccggccuguuu..................   254   0   Mm1
......................................................................aauugcacuugucccggccuguCu..................   1     1   Mm1
......................................................................aauugcacuugucccggGcuguuu..................   1     1   Mm1
......................................................................aauugcacuugucccggccuguAu..................   310   1   Mm1
......................................................................aauugcacuugucccggAcuguuu..................   1     1   Mm1
......................................................................aauugcacuugucccggccuguuA..................   1     1   Mm1
......................................................................aauugcacuuCucccggccuguuu..................   1     1   Mm1
......................................................................aauugcacuugucccggcAuguuu..................   1     1   Mm1
......................................................................aauugcUcuugucccggccuguuu..................   3     1   Mm1
......................................................................aauugcacuugucccggccuguuuU.................   14    1   Mm1
......................................................................aauugcacuugucccggccuguuuUa................   1     1   Mm1
.......................................................................auugcacuCgucccggccugu....................   26    1   Mm1
.......................................................................auugcacuugCcccggccugu....................   2     1   Mm1
.......................................................................auugcacuuguUccggccugu....................   1     1   Mm1
.......................................................................auugcacuuUucccggccugu....................   1     1   Mm1
.......................................................................auugcacuugucccggccugu....................   48    0   Mm1
.......................................................................auugcacuugucccggccugG....................   1     1   Mm1
.......................................................................auugcacuugucccggcGugu....................   1     1   Mm1
.......................................................................auugcacuugucccggccuguA...................   13    1   Mm1
.......................................................................auugcacuugucccggccuguu...................   3     0   Mm1
.......................................................................auugcacuCgucccggccuguu...................   1     1   Mm1
.......................................................................auugcacuugucccggccuguAu..................   14    1   Mm1
.......................................................................auugcacuugucccggccuguuu..................   3     0   Mm1
........................................................................uugcacuugucccggccugu....................   6     0   Mm1
........................................................................uugcacuugucccggcUugu....................   1     1   Mm1
..........................................................................gcacuugucccggccugu....................   1     0   Mm1

..................................aggcugugauAggugcaaaucu........................................................   4     1   Mm2
..................................aggcugugaugggugcaaaucu........................................................   3     0   Mm2
.....................................................................Caauugcacuugucccggccug.....................   1     1   Mm2
.....................................................................Caauugcacuugucccggccugu....................   39    1   Mm2
.....................................................................Uaauugcacuugucccggccugu....................   1     1   Mm2
.....................................................................Caauugcacuugucccggccuguu...................   2     1   Mm2
......................................................................aauugcacuugucccggc........................   2     0   Mm2
......................................................................aGuugcacuugucccggc........................   1     1   Mm2
......................................................................aauugcacuugAcccggcc.......................   1     1   Mm2
......................................................................aauugcacuugucccggcc.......................   38    0   Mm2
......................................................................aauugcaUuugucccggccu......................   1     1   Mm2
......................................................................aauugcacuuAucccggccu......................   4     1   Mm2
......................................................................aauugcacuugucccggAcu......................   2     1   Mm2
......................................................................aauugcacuugucccggccC......................   1     1   Mm2
......................................................................aauugcacuAgucccggccu......................   1     1   Mm2
......................................................................aauugcacuuCucccggccu......................   7     1   Mm2
......................................................................aauugcacuugucccggccu......................   313   0   Mm2
......................................................................aauugcacuugucccggccG......................   9     1   Mm2
......................................................................aauugcacuugucccggUcu......................   1     1   Mm2
......................................................................Uauugcacuugucccggccu......................   1     1   Mm2
......................................................................aauugcacuugucccggccuU.....................   7     1   Mm2
......................................................................aauugcacuugucccggccGg.....................   54    1   Mm2
......................................................................aauugcacCugucccggccug.....................   1     1   Mm2
......................................................................Cauugcacuugucccggccug.....................   1     1   Mm2
......................................................................aauugcacuugucccgUccug.....................   4     1   Mm2
......................................................................aauugcacuugucccgAccug.....................   9     1   Mm2
......................................................................aauugcGcuugucccggccug.....................   2     1   Mm2
......................................................................aauugcacuugucccggccuA.....................   2     1   Mm2
......................................................................aauugcacuugucccggGcug.....................   8     1   Mm2
......................................................................Uauugcacuugucccggccug.....................   3     1   Mm2
......................................................................aauugcacuugucccggccug.....................   1882  0   Mm2
```

```
                                   Star                              Mature
uuggaguuggauuuucacuugugucauguugggcaggcugugaugggugcaaaucuguaucaucaauuagaauugcacuugucccggccuguuugauugacugcaguucauc
......................................................................aauugcacuuguccUggccug.....................    1        1          Mm2
......................................................................aauugcacuugucccggccuC.....................    1        1          Mm2
......................................................................aauugcacuuAucccggccug.....................    3        1          Mm2
......................................................................aauugcacuugucccgCccug.....................    6        1          Mm2
......................................................................Nauugcacuugucccggccug.....................    1        1          Mm2
......................................................................aauugcacuugCcccggccug.....................    1        1          Mm2
......................................................................aauugcacuugucccggAcug.....................    25       1          Mm2
......................................................................aauugcacuugucccggUcug.....................    11       1          Mm2
......................................................................aauugcacuugucccggccCg.....................    1        1          Mm2
......................................................................aauugcacuugucAcggccug.....................    1        1          Mm2
......................................................................aauugcacuuCucccggccug.....................    1        1          Mm2
......................................................................aauugNacuugucccggccug.....................    1        1          Mm2
......................................................................aauugcacuuUucccggccug.....................    1        1          Mm2
......................................................................aauugcacuugucUcggccug.....................    3        1          Mm2
......................................................................aauugcacuAgucccggccugu....................    7        1          Mm2
......................................................................Uauugcacuugucccggccugu....................    97       1          Mm2
......................................................................aauugNacuugucccggccugu....................    13       1          Mm2
......................................................................aauugGacuugucccggccugu....................    2        1          Mm2
......................................................................aauugcaGuugucccggccugu....................    4        1          Mm2
......................................................................aauugUacuugucccggccugu....................    36       1          Mm2
......................................................................aauAgcacuugucccggccugu....................    10       1          Mm2
......................................................................aauugcacuugucccggccugu....................    113962   0          Mm2
......................................................................aauugcacuugucccggccuAu....................    12       1          Mm2
......................................................................aauugcacuuguGccggccugu....................    7        1          Mm2
......................................................................aauugcacuugucccgUccugu....................    226      1          Mm2
......................................................................aauugcacuugucccggccuUu....................    14       1          Mm2
......................................................................aauugcacuugucccAgccugu....................    25       1          Mm2
......................................................................aauCgcacuugucccggccugu....................    15       1          Mm2
......................................................................aUuugcacuugucccggccugu....................    27       1          Mm2
......................................................................aauugcacuugucccggcGugu....................    3        1          Mm2
......................................................................aauugcacuugGcccggccugu....................    2        1          Mm2
......................................................................aaGugcacuugucccggccugu....................    27       1          Mm2
......................................................................aauugcacGugucccggccugu....................    1        1          Mm2
......................................................................aauuUcacuugucccggccugu....................    17       1          Mm2
......................................................................aauugcacCugucccggccugu....................    13       1          Mm2
......................................................................Nauugcacuugucccggccugu....................    59       1          Mm2
......................................................................aauugcacuCgucccggccugu....................    6825     1          Mm2
......................................................................aauugcacuugucccggccuCu....................    50       1          Mm2
......................................................................aauugcacuugucccCgccugu....................    2        1          Mm2
......................................................................aauugcacuuguccGggccugu....................    5        1          Mm2
......................................................................aauugcaAuugucccggccugu....................    9        1          Mm2
......................................................................aauugcacuugAcccggccugu....................    14       1          Mm2
......................................................................aauugcacuuguccAggccugu....................    8        1          Mm2
......................................................................aCuugcacuugucccggccugu....................    11       1          Mm2
......................................................................aaAugcacuugucccggccugu....................    17       1          Mm2
......................................................................aauugcacuugucccggccugG....................    1539     1          Mm2
......................................................................aauugcUcuugucccggccugu....................    19       1          Mm2
......................................................................aauugcacuuCucccggccugu....................    108      1          Mm2
......................................................................aauugcacuugucccggcUugu....................    25       1          Mm2
......................................................................aauuAcacuugucccggccugu....................    21       1          Mm2
......................................................................aauugcacuugucccggccGgu....................    5        1          Mm2
......................................................................aauugcacuugucccgCccugu....................    295      1          Mm2
......................................................................aauugcacuuAucccggccugu....................    235      1          Mm2
......................................................................aauugcaUuugucccggccugu....................    18       1          Mm2
......................................................................Cauugcacuugucccggccugu....................    5        1          Mm2
......................................................................aauugcCcuugucccggccugu....................    3        1          Mm2
......................................................................aauugcacuugucAcggccugu....................    31       1          Mm2
......................................................................aauugAacuugucccggccugu....................    8        1          Mm2
......................................................................aauugcacuugucccggccugC....................    204      1          Mm2
......................................................................aauugcNcuugucccggccugu....................    7        1          Mm2
......................................................................aauuCcacuugucccggccugu....................    11       1          Mm2
......................................................................aauugcacuuUucccggccugu....................    113      1          Mm2
......................................................................aauugcGcuugucccggccugu....................    39       1          Mm2
......................................................................aauGgcacuugucccggccugu....................    5        1          Mm2
......................................................................aGuugcacuugucccggccugu....................    21       1          Mm2
......................................................................aaCugcacuugucccggccugu....................    15       1          Mm2
......................................................................aauugcacuugucccggccugA....................    46       1          Mm2
......................................................................aauugcacuuguccUggccugu....................    40       1          Mm2
......................................................................Gauugcacuugucccggccugu....................    19       1          Mm2
......................................................................aauugcacuugucUcggccugu....................    60       1          Mm2
```

```
                                  Star                                Mature
uuggaguuggauuuucacuugugucauguugggcaggcugugaugggugcaaaucuguaucaucaauuagaauugcacuugucccggccuguuugauugacugcaguucauc
......................................................................aauugcacuugucGcggccugu....................  13    1  Mm2
......................................................................aauugcacAugucccggccugu....................  8     1  Mm2
......................................................................aauugcacuGgucccggccugu....................  7     1  Mm2
......................................................................aauugcacuugucccggccAgu....................  5     1  Mm2
......................................................................aauugcacuugucccggcAugu....................  5     1  Mm2
......................................................................aauugcacuuguAccggccugu....................  11    1  Mm2
......................................................................aauugcacuugCcccggccugu....................  11    1  Mm2
......................................................................aauugcacuugucccggccCgu....................  16    1  Mm2
......................................................................aauugcacuugucccgAccugu....................  293   1  Mm2
......................................................................aauugcacuugucccUgccugu....................  14    1  Mm2
......................................................................aauugcacuugucccggUcugu....................  589   1  Mm2
......................................................................aauugcacuugucccggGcugu....................  411   1  Mm2
......................................................................aauugcacuugucccggAcugu....................  1015  1  Mm2
......................................................................aauugcacuuguUccggccugu....................  48    1  Mm2
......................................................................aNuugcacuugucccggccugu....................  1     1  Mm2
......................................................................aauugcacuugucccCgccuguu...................  1     1  Mm2
......................................................................aauugcacuugucccggccuUuu...................  2     1  Mm2
......................................................................aauugcacuuAucccggccuguu...................  22    1  Mm2
......................................................................aauugcacuugucccggccugCu...................  3     1  Mm2
......................................................................aauugcacuuUucccggccuguu...................  8     1  Mm2
......................................................................aauugcacuugucccgCccuguu...................  27    1  Mm2
......................................................................aauugcacuugucccggccuCuu...................  2     1  Mm2
......................................................................aauugNacuugucccggccuguu...................  3     1  Mm2
......................................................................aauuUcacuugucccggccuguu...................  1     1  Mm2
......................................................................aauugcacuugucccggccuguG...................  35    1  Mm2
......................................................................aauugcacuugucccAgccuguu...................  1     1  Mm2
......................................................................Uauugcacuugucccggccuguu...................  5     1  Mm2
......................................................................Gauugcacuugucccggccuguu...................  1     1  Mm2
......................................................................aauugcacuugucccggccCguu...................  1     1  Mm2
......................................................................aauugcacuugucccggcUuguu...................  2     1  Mm2
......................................................................aauugUacuugucccggccuguu...................  2     1  Mm2
......................................................................aauugcacuCgucccggccuguu...................  372   1  Mm2
......................................................................aauugcaUuugucccggccuguu...................  2     1  Mm2
......................................................................aauugcacuugucAcggccuguu...................  3     1  Mm2
......................................................................aauugcacuugucccgAccuguu...................  32    1  Mm2
......................................................................aauugcacuugucccggcAuguu...................  1     1  Mm2
......................................................................aauugcacuugucccggGcuguu...................  29    1  Mm2
......................................................................aauugcacuuguUccggccuguu...................  4     1  Mm2
......................................................................aauugcacuugucccggccuguA...................  3949  1  Mm2
......................................................................aauugcUcuugucccggccuguu...................  1     1  Mm2
......................................................................aauugcGcuugucccggccuguu...................  3     1  Mm2
......................................................................aauugcacuuguccUggccuguu...................  8     1  Mm2
......................................................................aaAugcacuugucccggccuguu...................  1     1  Mm2
......................................................................aauugcacuugAcccggccuguu...................  1     1  Mm2
......................................................................aaGugcacuugucccggccuguu...................  2     1  Mm2
......................................................................aauugAacuugucccggccuguu...................  3     1  Mm2
......................................................................aUuugcacuugucccggccuguu...................  2     1  Mm2
......................................................................aauAgcacuugucccggccuguu...................  3     1  Mm2
......................................................................aauugcacuAgucccggccuguu...................  1     1  Mm2
......................................................................aauugcacuuCucccggccuguu...................  5     1  Mm2
......................................................................aauugcacuugucccggUcuguu...................  45    1  Mm2
......................................................................aauugcacuugucccggAcuguu...................  81    1  Mm2
......................................................................Nauugcacuugucccggccuguu...................  2     1  Mm2
......................................................................aauugcacuugucccggccugAu...................  1     1  Mm2
......................................................................aauugcacuugucUcggccuguu...................  6     1  Mm2
......................................................................aauCgcacuugucccggccuguu...................  4     1  Mm2
......................................................................aauugcacuugucccggccugGu...................  3     1  Mm2
......................................................................aauugcacuugucccggccuguu...................  9002  0  Mm2
......................................................................aauugcacuuguccAggccuguu...................  3     1  Mm2
......................................................................aauugcacuugucccggccuguC...................  9     1  Mm2
......................................................................aauugcacuugCcccggccuguu...................  1     1  Mm2
......................................................................aauugcacuugucccgUccuguu...................  18    1  Mm2
......................................................................aauugcacuugucccggccuUuuu..................  2     1  Mm2
......................................................................aauugcaAuugucccggccuguuu..................  2     1  Mm2
......................................................................aauugcacuCgucccggccuguuu..................  86    1  Mm2
......................................................................aauugcacuuCucccggccuguuu..................  1     1  Mm2
......................................................................aauugcacuugucccggccuCuuu..................  1     1  Mm2
......................................................................Nauugcacuugucccggccuguuu..................  2     1  Mm2
......................................................................Uauugcacuugucccggccuguuu..................  2     1  Mm2
......................................................................aauugcacuugucccggccuguCu..................  7     1  Mm2
```

**Star** **Mature**

```
uuggaguuggauuuucacuugugucauguugggcaggcugugaugggugcaaaucuguaucaucaauuagaauugcacuugucccggccuguuugauugacugcaguucauc
......................................................................aauugcacuugucccggccuguGu..................  2     1  Mm2
......................................................................aauugcacuugucccggUcuguuu..................  9     1  Mm2
......................................................................aauugcacuugucccggccuguuG..................  4     1  Mm2
......................................................................aauugcacuugucccgAccuguuu..................  7     1  Mm2
......................................................................aaAugcacuugucccggccuguuu..................  1     1  Mm2
......................................................................aauugcacuugucUcggccuguuu..................  2     1  Mm2
......................................................................aauugcacuuUucccggccuguuu..................  3     1  Mm2
......................................................................aauugcacuugucccggccuguuu..................  1557  0  Mm2
......................................................................aauugcaUuugucccggccuguuu..................  1     1  Mm2
......................................................................aauugcacuGgucccggccuguuu..................  1     1  Mm2
......................................................................aauugcacuugucccggccCguuu..................  1     1  Mm2
......................................................................aauuAcacuugucccggccuguuu..................  1     1  Mm2
......................................................................aauCgcacuugucccggccuguuu..................  1     1  Mm2
......................................................................aauugcacuuAucccggccuguuu..................  4     1  Mm2
......................................................................aauugcacuuguGccggccuguuu..................  1     1  Mm2
......................................................................aauugcacuugucccggccuguAu..................  1211  1  Mm2
......................................................................aauugcacuugucccgUccuguuu..................  4     1  Mm2
......................................................................aauugcacuugucccggGcuguuu..................  5     1  Mm2
......................................................................aauugcacuugucccggccuguuA..................  48    1  Mm2
......................................................................aauugcacuugucccggccuguuC..................  5     1  Mm2
......................................................................aauugcacuugucccggAcuguuu..................  9     1  Mm2
......................................................................aauugcacuugucccggccuguuug.................  2     0  Mm2
......................................................................aauugcacuugucccggccuguuuA.................  1     1  Mm2
......................................................................aauugcacuugucccggccuguuuU.................  140   1  Mm2
......................................................................aauugcacuugucccggccuguuuC.................  1     1  Mm2
......................................................................aauugcacuugucccggccuguAug.................  1     1  Mm2
.......................................................................auugcacuugucccggccug.....................  8     0  Mm2
.......................................................................auugcacuuguUccggccug.....................  1     1  Mm2
.......................................................................auugcacuugGcccggccugu....................  2     1  Mm2
.......................................................................auuAcacuugucccggccugu....................  2     1  Mm2
.......................................................................auugcacuugucccggccugG....................  3     1  Mm2
.......................................................................auugcacuugucccggccuUu....................  2     1  Mm2
.......................................................................auugcacuGgucccggccugu....................  1     1  Mm2
.......................................................................auugcacuCgucccggccugu....................  22    1  Mm2
.......................................................................auugcacuuguUccggccugu....................  2     1  Mm2
.......................................................................auugcacuugucccggccugu....................  201   0  Mm2
.......................................................................auuUcacuugucccggccugu....................  1     1  Mm2
.......................................................................auugGacuugucccggccugu....................  1     1  Mm2
.......................................................................auugcacuugucccggccCgu....................  2     1  Mm2
.......................................................................auugcacuugucccggAcugu....................  1     1  Mm2
.......................................................................auugcacuuUucccggccugu....................  5     1  Mm2
.......................................................................auugcacuuUucccggccuguu...................  1     1  Mm2
.......................................................................auugcacuugucccggccuguA...................  42    1  Mm2
.......................................................................auugcacuugucccggccuguu...................  20    0  Mm2
.......................................................................auugcacuCgucccggccuguu...................  2     1  Mm2
.......................................................................auuUcacuugucccggccuguu...................  1     1  Mm2
.......................................................................auugcacuugucccggccuguAu..................  10    1  Mm2
.......................................................................auugcacuugucccggccuguuu..................  6     0  Mm2
.......................................................................auugcacuugucccggccuguuA..................  1     1  Mm2
.......................................................................auugcacuCgucccggccuguuu..................  2     1  Mm2
........................................................................uugcacuugucccggccugu....................  4     0  Mm2
.........................................................................ugcacuugucccggccugu....................  3     0  Mm2
.........................................................................ugcacuugucccggccuCu....................  1     1  Mm2
..........................................................................gcacuugucccggccugu....................  4     0  Mm2
```
